# Ablation of paroxysmal and persistent atrial fibrillation in the very elderly real‐world data on safety and efficacy

**DOI:** 10.1002/clc.23485

**Published:** 2020-10-19

**Authors:** Kevin Willy, Kristina Wasmer, Dirk G. Dechering, Julia Köbe, Philipp S. Lange, Nils Bögeholz, Christian Ellermann, Florian Reinke, Gerrit Frommeyer, Lars Eckardt

**Affiliations:** ^1^ Department of Cardiology II–Electrophysiology University Hospital Münster Münster Germany

## Abstract

**Background:**

The role and technique of catheter ablation of atrial fibrillation (AF) in the elderly is unclear. While in young patients pulmonary vein isolation (PVI) has evolved as first option, in older patients decision is often made in favor of drugs as higher complication rates and less benefit are suspected. Therefore, data on PVI of paroxysmal and persistent AF in these patients is still sparse but of eminent importance.

**Hypothesis:**

PVI is comparably safe in the very elderly with similar recurrence and complication rates.

**Methods:**

We enrolled all patients (n = 146) aged >75 years who underwent a first PVI over a period of 10 years (2009‐2019) from our prospective single‐center ablation registry. Mean follow‐up time was 231 ± 399 days.

**Results:**

Acute ablation success defined as complete PVI and sinus rhythm at the end of the ablation procedure was high (99%). Severe periprocedural complications occurred in 3.3% (stroke/TIA n = 2; 1.3%; pericardial effusion n = 3; 2%). In 4.6% of patients symptomatic sick‐sinus‐syndrome was unmasked after PVI resulting in pacemaker implantation. There were no deaths related to PVI. Recurrence rate of symptomatic AF was 37.3% resulting in a Re‐PVI and/or substrate ablation in 32 pts (20.9%). During follow‐up pacemaker implantation plus atrioventricular node ablation was performed in 10 pts (6.8%). There was a trend toward lower recurrence rates with single‐shot devices (cryoballoon, multielectrode phased‐radiofrequency ablation catheter) than with point‐by‐point radiofrequency while complication rates did not differ.

**Conclusion:**

PVI for AF is a feasible treatment option also in patients >75 years with a reasonable success and safety profile. Higher success rates occurred in patients treated with a single‐shot device as compared to point‐by‐point ablation.

## 1 INTRODUCTION

Atrial fibrillation (AF) is the most common atrial arrhythmia. With increasing age AF is becoming clinically manifest in a growing number of patients. In parallel, the need and wish for definitive therapy is growing due to improved ablation techniques with higher success and low‐complication rates. Catheter ablation has developed as first line therapy in various arrhythmias. This is reflected in a strengthening of ablation in the recently updated ESC guidelines for the management of supraventricular tachycardias.^1^ Interestingly, recommendations are not age‐dependent. In AF, however, many centers have an individual age limit for ablation.^2^

Pulmonary vein isolation (PVI) has been developed as the cornerstone of AF ablation. Specific data on AF ablation of patients >75 years was first published by Nademannee et al.^3^ in 2015. A recent multicentre study presented high‐success rates in 104 patients ≥75 years who underwent PVI with the cryoballoon.^4^ In order to compare a “single‐shot” strategy such as the cryoballoon to a point‐by‐point ablation approach we performed an analysis of patients >75 years undergoing their first PVI.

## 2 MATERIAL AND METHODS

The study was conducted in accordance with the guidelines of the Declaration of Helsinki. In the present study, we analyzed our prospective single‐center database for a period of 10 years (2009‐2019). We included all patients >75 years who underwent their first PVI for drug‐refractory highly symptomatic AF. Acute success rates, complications, recurrence rates, redo procedures, and AF therapy during follow‐up were recorded.

### 2.1 Procedure of catheter ablation

Every patient signed written informed consent prior to the ablation procedure. Transesophageal echocardiography was performed immediately prior to the procedure in all patients. PVI was done with the cryoballon (second‐generation cryoballoon [ArcticFront Advance, Medtronic, Minneapoiis]; n = 79), the multielectrode phased‐radiofrequency ablation catheter (PVAC Gold catheter [Medtronic, Minneapolis, USA], n = 41)^5^ or a radiofrequency point‐by‐point ablation (n = 26)^6^ under sedation with midazolam and/or propofol. Surface electrocardiograms and endocardial electrograms were continuously monitored and stored on a computer‐based recording system. Patients of cryoballoon and the PVAC group were treated with one transseptal sheath. Patients of the radiofrequency (RF)‐group were ablated employing two transseptal sheaths for a decapolar LASSO‐catheter and a 3.5 mm irrigated tip catheter (Tacticath, St. Jude Medical, Saint Paul, Minnesota) and a 3D mapping system (NavX, St. Jude Medical).^7^ In all groups, the catheter setup was complemented by a decapolar coronary sinus catheter and a quadripolar catheter that was positioned in the right ventricle. After ablation, protone pump‐inhibitors were added to the medication of every patient for 4 weeks after ablation to prevent esophageal damage associated to ablation.^8^, ^9^, ^10^

### 2.2 Statistical analysis

Continuous data are reported as mean ± SD, categorical data are reported as percentages. Statistical analysis was performed using GraphPad PRISM 6.0 (San Diego, California) and the SPSS Statistics, version 20.0 (SPSS, Inc., Chicago). A *P*‐value < .05 was considered statistically significant.

## 3 RESULTS

### 3.1 Acute outcomes

There were 146 consecutive patients over 75 years receiving a first PVI in our clinic between 08/2009 and 09/2019 with an established diagnosis of either symptomatic paroxysmal (46.6%) or persistent (53.4%) AF. Acute success defined as complete PVI and sinus rhythm at the end of the procedure was achieved in all but one patient (99.3%). In 54.1% PVI was performed using the cryoballoon technique whereas 45.9% where ablated using RF energy (point‐by‐point ablation n = 26; 17.8% and PVAC n = 41; 28.1%). 49.7% of patients were ablated during sinus rhythm, while 50.3% had AF before ablation. In 47.9% of patients an electrical cardio version was performed during the ablation procedure. Prior to ablation, 11.8% already had had ablation of the cavotricuspidal isthmus for atrial flutter. In 4.8% of patients undergoing PVI in additional cavotricuspid isthmus (CTI) ablation was performed because of documented atrial flutter.

### 3.2 Baseline data and demographics

Concerning baseline characteristics, no significant differences were observed if grouped for ablation device. There were significant differences between patients with paroxysmal and persistent AF regarding gender, LA size, and structural heart disease (Table 1A). The mean duration from first diagnosis of AF to ablation was 5.3 ± 4.9 years. Mean EHRA stadium was 2.7 ± 0.5 while mean CHA_2_DS_2_‐VASc‐score was 3.9 ± 1.0. The majority of patients (66%) had no evidence of structural heart disease while among the patients with structural heart diseases ischemic cardiomyopathy was most common (19.2%). A history of tachycardiomyopathy was present in 12 pts (8.2%). All but one patient with a Left Atrial Appendage (LAA) occluder were took oral anticoagulation (42.5% vitamin K antagonists, 57.5% direct oral anticoagulants).

**TABLE 1A:** Patient charateristics at baseline grouped according to the ablation device

	All patients (n = 146)	Cryo (n = 79)	RF (PVAC) (n = 41)	RF(3 D) (n = 26)
Age	77.8 ± 2.3	76.9 ± 9	78.0 ± 2.2	77.1 ± 2.6
Male	73 (50%)	47 (56.7%)	14 (34.1%)^a^	18 (62.1%)
Arterial hypertension	127 (87%)	69 (87.3%)	33 (80.5%)	28 (96.6%)
Diabetes mellitus	18 (12.3%)	7 (8.9%)	7 (17.1%)	5 (17.2%)
Structural heart disease	45 (34%)	23 (29.1%)	14 (34.1%)	8 (27.6%)
0 = normal LA size	21 (14.4%)	11 (13.9%)	8 (19.5%)	2 (7.7%)
Dilatation				
1°	63 (43.2%)	35 (44.3%)	18 (43.9%)	10 (38.5%)
2°	34 (23.2%)	15 (19%)	8 (19.5%)	11 (42.3%)
3°	28 (19.2%)	18 (22.8%)	7 (17.1%)	3 (11.5%)
LV‐EF (%) LV‐EF <50%	56.6 25 (17.1%)	55.9 15 (19%)	56.6 7 (17.1%)	58.3 2 (7.7%)
CHA_2_DS_2_‐VASc‐score	3.9 ± 1.0	3.6 ± 1.0	4.2 ± 1.0	4.0 ± 0.9
EHRA stadium (I‐IV)	2.7 ± 0.5	2.5 ± 0.5	2.8 ± 0.6	2.9 ± 0.4
Duration from diagnosis to ablation (y)	5.3 ± 4.9	4.9 ± 4.7	4.7 ± 4.5	7.5 ± 5.5
BMI (kg/m^2^)	26.3 ± 3.4	26.1 ± 3.1	26.7 ± 4.0	26.5 ± 3.0

*Note*: Data are expressed as numbers with percentages or mean with SD.

Abbreviations: AF, atrial fibrillation; BMI, body mass index; Cryo, cryoballoon; EHRA, European Heart Journal Association classification of AF symptoms; LA, left atrium; RF, radiofrequency.

^a^ Significant difference compared to reference group (cryoablation group) (*P* < .05).

### 3.3 Follow‐up and complications

In patients being ablated with the cryoballoon, recurrence rate was 29.1%, with PVAC 36.6%, and for patients with a 3D mapping guided PVI 46.2% (*P* = .11, n.s.). In turn, mean follow‐up duration was shorter in patients being ablation with cryo (162 days) than with PVAC (340 days) or 3D RF (249 days) (Table 1A and 2A).

Regarding the type of AF, in patients with paroxysmal AF there was a recurrence rate of 29.9% while recurrence rate was 48.7% (*P* < .05) in patients with persistent AF in the presence of comparable follow‐up duration (Table 1B and 2B).

**TABLE 1B:** Patient charateristics at baseline grouped according to the type of AF

	All patients (n = 146)	Paroxysmal AF (n = 68)	Persistent AF (n = 78)
Age	77.8 ± 2.3	78.0 ± 2.4	77.6 ± 2.2
Male	73 (50%)	30 (44.1%)	49 (57.6%)^a^
Arterial hypertension	127 (87%)	56 (82.4%)	72 (92.3%)
Diabetes mellitus	18 (12.3%)	11 (16.2%)	7 (9.0%)
Structural heart disease	45 (34%)	13 (19.1%)	33 (38.8%)^a^
0 = normal LA size	21 (14.4%)	16 (23.5%)	6 (7.7%)^a^
Dilatation			
1°	63 (43.2%)	35 (51.5%)	28 (35.9%)^a^
2°	34 (23.2%)	11 (16.2%)	23 (29.5%)^a^
3°	28 (19.2%)	6 (8.8%)	22 (28.2%)^a^
LV‐EF (%) LV‐EF <50%	56.6 25 (17.1%)	58.7 3 (4.4%)	55.2 22 (25.9%)^a^
CHA_2_DS_2_‐VASc‐score	3.9 ± 1.0	3.8 ± 1.0	3.9 ± 0.9
EHRA stadium (I‐IV)	2.7 ± 0.5	2.6 ± 0.6	2.7 ± 0.5
Duration from diagnosis to ablation (years)	5.3 ± 4.9	5.1 ± 4.9	5.5 ± 4.8
BMI (kg/m^2^)	26.3 ± 3.4	26.3 ± 3.2	26.2 ± 3.5

*Note*: Data are expressed as numbers with percentages or mean with SD.

Abbreviations: AF, atrial fibrillation; BMI, body mass index; Cryo, cryoballoon; EHRA, European Heart Journal Association classification of AF symptoms; LA, left atrium; RF, radiofrequency.

^a^ Significant difference between paroxysmal and persistent AF (*P* < .05).

**TABLE 2A:** Results grouped according to the ablation device

	All patients (n = 146)	Cryo (n = 79)	RF (PVAC) (n = 41)	RF (3D) (n = 26)
Acute ablation success	145 (99.3%)	78 (98.7%)	41 (100%)	26 (100%)
Free from recurrency during follow‐up	89 (62.7%)	56 (70.9%)	26 (63.4%)	14 (53.8%)
Re‐AF ablation during follow‐up	32 (20.9%)	7 (8.9%)	11 (26.8%)^a^	6 (23%)^a^
AVN ablation during follow‐up	10 (6.8%)	1 (1.3%)	6 (14.6%)^a^	3 (11.5%)^a^
Reconnection of PV during second PVI				
LSPV	20 (13.7%)	4 (5.1%)	7 (17.1%)	4 (15.4%)
LIPV	16 (11.0%)	3 (3.8%)	7 (17.1%)	2 (7.7%)
RSPV	18 (12.3%)	4 (5.1%)	4 (9.8%)	5 (19.2%)
RIPV	18 (12.3%)	5 (6.3%)	5 (12.2%)	5 (19.2%)
Additional LA ablation lines	2 (1.4%)	0 (0%)	0 (0%)	2 (7.7%)
Additional CTI ablation	7 (4.8%)	3 (3.8%)	4 (9.7%)	0 (0%)
Overall complications	8 (5.5%)	4 (5.2%)	3 (7.3%)	1 (3.8%)
Major complications	5 (3.4%)	2 (2.6%)	2 (4.8%)	1 (3.8%)
Cerebrovascular Pericardial effusion	2 (1.4%) 3 (2.0%)	1 (1.3%) 1 (1.3%)	1 (2.4%) 1 (2.4%)	1 (3.8%)
Minor Vascular without surgery Transient phrenic palsy	3 (2.1%) 2 (1.4%) 1 (0.7%)	2 (2.6%) 1 (1.3%) 1 (1.3%)	1 (2.4%) 1 (2.4%)	0 (0%)
PM implantation for SSS	6 (4.1%)	4 (5.1%)	1 (2.4%)	1 (3.8%)

*Note*: Data are expressed as numbers with percentages or mean with SD.

Abbreviations: AF, atrial fibrillation; AVN, atrioventricular node; Cryo, cryoballoon; CTI, cavotricuspid isthmus; LA, left atrium; LIPV, left inferior pulmonary vein; LSPV, left superior pulmonary vein; PM, pacemaker; RF, radiofrequency; RIPV, right inferior pulmonary vein; RSPV, right superior pulmonary vein; SSS, sick‐sinus‐syndrome.

^a^ Significant difference compared to reference group (cryoablation group) (*P* < .05).

**TABLE 2B:** Results grouped according to the type of AF

	All patients (n = 146)	Paroxysmal AF (n = 68)	Persistent AF (n = 78)
Acute ablation success	145 (99.3%)	68 (100%)	87 (98.7%)
Free from recurrency during follow‐up	89 (62.7%)	48 (70.1%)	40 (51.3%)^a^
Re‐AF ablation during follow‐up	32 (20.9%)	13 (19.1%)	20 (25.6%)
AVN ablation during follow‐up	10 (6.8%)	4 (5.9%)	6 (7.1%)
Reconnection of PV during second PVI			
LSPV	20 (13.7%)	8 (11.8%)	12 (15.3%)
LIPV	16 (11.0%)	7 (10.3%)	9 (11.5%)
RSPV	18 (12.3%)	8 (11.8%)	11 (14.1%)
RIPV	18 (12.3%)	8 (11.8%)	11 (14.1%)
Additional LA ablation lines	2 (1.4%)	2 (2.9%)	0 (0%)
Additional CTI ablation	7 (4.8%)	1 (1.4%)	6 (7.7%)
Overall complications	8 (5.5%)	3 (4.4%)	6 (7.2%)
Major complications Cerebrovascular Pericardial effusion	5 (3.4%) 2 (1.4%) 3 (2.0%)	2 (3.0%) 1 (1.5%) 1 (1.5%)	3 (3.8%) 1 (1.3%) 2 (2.5%)
Minor Vascular without surgery Transient phrenic palsy	3 (2.1%) 2 (1.4%) 1 (0.7%)	1 (1.4%) 1 (1.4%)	3 (3.8%) 2 (2.5%) 1 (1.3%)
PM implantation for SSS	6 (4.1%)	3 (4.4%)	3 (3.8%)

*Note*: Data are expressed as numbers with percentages or mean with SD.

Abbreviations: AF, atrial fibrillation; AVN, atrioventricular node; Cryo, cryoballoon; CTI, cavotricuspid isthmus; LA, left atrium; LIPV, left inferior pulmonary vein; LSPV, left superior pulmonary vein; PM, pacemaker; RF, radiofrequency; RIPV, right inferior pulmonary vein; RSPV, right superior pulmonary vein; SSS, sick‐sinus‐syndrome.

^a^ Significant difference between paroxysmal and persistent AF (*P* < .05).

During follow‐up, a “pace‐and‐ablate” therapy for recurrent symptomatic AF with ablation of the atrioventricular node (AVN) was performed in 6.8% of patients.

Major complications (pericardial effusion, stroke, ICU treatment, vascular complications requiring surgical treatment) occurred in 3.3% of patients (2% pericardial effusion, 1.3% stroke). Overall complications occurred in 5.9% of patients.

After ablation symptomatic sick‐sinus‐syndrome was unmasked in 4.1% of patients who were then implanted with a permanent pacemaker during the same stay. There were no adverse events recorded regarding pacemaker implantation.

## 4 DISCUSSION

In this study, we present data on AF ablation in elderly patients aged over 75 years. We found success rates comparable to those reported in literature with recurrence rates around 30% to 50% during follow‐up and low‐overall complication rates. As a novel finding, cryoballoon ablation as well as multielectrode phased‐radiofrequency ablation tended to be more effective and had similar complication rates compared to conventional RF ablation using 3D mapping systems without reaching statistical significance, mostly due to the low number of patients ablated with RF. A trend toward better results of PVI with the cryoballoon in the elderly patient cohort has already discussed in literature.^11^ Heeger et al.^4^ reported data for 104 patients ablated with the second‐generation cryoballoon and showed low‐recurrence rates of 20% after 1 year and 40% after 3 years of follow‐up with a single ablation procedure in patients over 75 years. In addition, Metzner et al.^12^ published 94 patients >75 years ablated with RF. In a mean follow‐up of 37 months only 38% of patients remained in sinus rhythm with a single ablation procedure. With repeated ablation procedures the number of patients in SR could be raised to 59%. These two studies underline the high‐overall safety and the reasonable success rates which we are also observed in our data on the one hand as well as the difference in efficacy between the two energy forms in this patient collective. In our analysis, we found mid‐term success rates of the multielectrode phased‐radiofrequency ablation comparable to those achieved with the cryoballoon so that possibly not only the form of energy delivered but also differences to point‐by‐point ablation and single‐shot ablation devices might play a role. Furthermore, complications due to consecutive left atrial tachycardias as a result of the PVI have been demonstrated to be lower in patients ablated with multielectrode phased‐radiofrequency ablation than with point‐by‐point RF.^13^ An enhanced safety profile of single‐shot‐ablation devices such as multielectrode phased‐radiofrequency ablation and cryoballoon in comparison the conventional RF ablation could also be underlined by De Greef et al.^14^ Results of the multielectrode phased‐radiofrequency ablation was favorable at least in a mixed cohort of almost 400 patients with paroxysmal or persistent AF.^15^ These differences might be an expression of the different learning curves for ablation techniques. While in cryoballon trials the success rates are mostly comparable, the results from RF trials differ to a higher extent possibly because they are more dependent on experience and skill of the operator.

In contrast to the trial by Metzner et al,^12^ we included a larger number of patients with persistent AF, which might have resulted in a higher recurrence rate in our trial. This is even more important as Santangeli et al^16^ demonstrated that especially in very elderly patients AF triggers were often not only localized in the pulmonary veins. As in persistent AF the substrate is often more complex and located outside the pulmonary veins resulting in higher recurrence rates. One may speculate that a more extensive ablation approach may result in higher long‐term success rates in elderly patients although long procedure times and accordingly rising risks of complications might be limiting. Surprisingly, in a trial by Nademannee et al^3^ elimination of CFAEs without PVI led to a very high rate of patients (83%) maintaining sinus rhythm in a follow‐up of about 3 years. They could also show that keeping patients in sinus rhythm was associated with a lower mortality in this patient group. In this trial, patients on NOACs were excluded, symptomatology was not assessed and played no major role in decision pro or contra ablation and many patients were ablated although they had long‐standing persistent AF. Furthermore, PVI was not performed in this study but only CFAE ablation, which hampers comparability as PVI is nowadays the cornerstone of every AF ablation. Bunch et al^17^, however, found no difference concerning the success rates of RF ablation for AF in octogenarians compared to a younger control group.

Furthermore, we observed that there was only a limited number of patients with subsequent AVN ablation. Evidence on the outcome of elderly patients undergoing AVN ablation is sparse and not clear. While Wasmer et al^18^ could demonstrate a similar symptom relief in patients with AVN ablation compared with PVI and a lower rate of rehospitalisation despite higher age and more comorbidities, Eitel et al^19^ reported data from the German ablation registry which revealed an increased mortality when opting for AVN ablation but not when choosing PVI in patients with heart failure and reduced ejection fraction.

### 4.1 Risk of peri‐ and post‐procedural complications

Major complications only occurred in 3.3% of patients ‐ 1.3% with cerebrovascular events, 2% with pericardial effusion. This is in line or even slightly below the complication rates described in previous studies^4^, ^12^ and comparable to data from the world‐wide survey of AF ablation^20^ and to large prospective randomized controlled trials such as the fire‐and‐ice trial.^21^ However, Guiot et al^22^ demonstrated that age > 75 years is an independent predictor of late cerebrovascular events after ablation of AF so that the rate might be higher when extending the follow‐up duration. Nevertheless, AF ablation has also be shown to potentially reduce cerebrovascular events in patients with AF^23^, ^24^ so that it might be worth taking the risk. However, those positive results mainly stem from registries while randomized trials have failed to show a significant reduction according to a recent meta‐analysis by Barra et al.^25^

## 5 CONCLUSION

Catheter ablation of AF in patients ≥75 years of age is associated with a good safety profile and a favorable clinical outcome in patients with paroxysmal as well as persistent AF. The data is in favor of the single‐shot devices such as the cryoballoon for PVI in this patient cohort. Randomized trials have to be performed to further evaluate this possible advantage. Only few patients are in need for an AVN ablation during follow‐up.

## Data Availability

The data that support the findings of this study are available from the corresponding author upon reasonable request.
